# Airway stenting with the LT-Mold™ for severe glotto-subglottic stenosis or intractable aspiration: experience in 65 cases

**DOI:** 10.1007/s00405-012-2080-x

**Published:** 2012-06-22

**Authors:** Jaber Alshammari, Philippe Monnier

**Affiliations:** Department of Otorhinolaryngology, Head and Neck Surgery, University Hospital CHUV, Lausanne, Switzerland

**Keywords:** Laryngotracheal stenosis, Airway stenting, LT-Mold, Laryngotracheal reconstruction, Partial cricotracheal resection, Intractable aspiration

## Abstract

The purpose of this study was to assess the safety and efficacy of stenting in upper airway reconstructions for benign laryngotracheal stenosis (LTS) with a newly designed prosthesis, the LT-Mold™. The LT-Mold and its proper use during open surgery and endoscopy are described, and the experience gathered from a prospectively collected database on 65 patients treated for complex LTS or severe aspiration is reported. This series is compared to the results of other stenting methods. All patients were available for evaluation. In all but one case, the prosthesis was removed at the end of the study. The new prosthesis did not induce any stent-related trauma to the supraglottis, glottis and subglottis. Before adding a distal round-shaped silicone cap to the LT-Mold, granulation tissue was usually seen at the stent-mucosal interface at the tracheostoma level. In 14 cases, there has been a spontaneous extrusion of the prosthesis through the mouth; this problem was solved by fixing the prosthesis through the reinforced portion of the prosthesis at the cap level and by adding one fixation stitch in the supraglottis. We have to document the loss of the silicone cap in three cases. This problem was resolved by designing a new prototype with an integrated cap, glued with a slow hardening silicone glue. Fifty-four (83 %) of 65 patients were decannulated after a mean duration of stenting of 3 months (range 1–12 months). The mean follow-up after decannulation was 23 months (range 1 month to 10 years). The experience gathered with the LT-Mold shows that long-term stenting for complex LTS is safely achieved when the prosthesis is used with its distal integrated silicone cap. The softness and smoothness of the prosthesis with a round-shaped configuration of both extremities help avoid ulceration and granulation tissue formation in the reconstructed airway. Adequate fixation is mandatory to avoid extrusion.

## Introduction

With modern surgical techniques such as partial cricotracheal resection (PCTR), even the most severe grades of isolated subglottic stenoses (SGS) can be treated without cartilage grafting and stenting [1, 2]. However, when the cicatricial SGS extends cranially to the glottis or supraglottis, stenting of the airway in addition to surgery is necessary for several reasons. These include stabilization of the posterior cartilage graft used for the cure of a posterior glottic stenosis (PGS), prevention of the recurrence of a web after division of a cicatricial fusion of the vocal cords and splinting of a complex airway reconstruction when the larynx is distorted after previously failed laryngotracheal reconstruction (LTR) [3–5].

Laryngeal stents are mainly used to keep the airway expanded after surgical reconstruction or trauma. They provide support and immobilization of cartilage grafts and mucosal flaps to the recipient site and maintain the lumen in the reconstructed airway that temporarily lacks adequate support. Unfortunately, laryngeal stents can also act as foreign bodies in the reconstructed airway and induce mucosal injuries, ulcerations, granulation tissue formation and subsequent restenosis if their anatomical conformity to the inner laryngeal contours is not perfect or if their consistency is too hard. Ideally, a dedicated laryngeal stent should conform to airway contours, exert <30 mmHg of mucosal pressure, resist compressive forces, maintain airway anatomy, move with the larynx during respiration and deglutition and be biocompatible [6].

Several laryngeal stents are currently available on the market. The simplest ones, namely the finger cot and the rolled silastic sheet are custom made [7]. They are quite primitive and have now been largely replaced by the Aboulker stent [8–10], the Montgomery T-tube [11], the Healy—Montgomery pediatric T-tube and the Montgomery [12] or Eliachar laryngotracheal stents [13]. Unfortunately, none of these stents truly meets the aforementioned requirements for safe use without potential damage to the reconstructed airway. As stenting is still unavoidable after complex airway reconstructions involving the glottis, it is astonishing that the shape of most currently available stents has remained quite primitive in comparison with the complexity of the inner laryngeal contours. If stents had fewer adverse effects on the healing process after an LTR or an extended PCTR, they could be used with less caution and improved results for solving the most difficult cases of airway stenosis in infants, children and adults.

This study presents the latest refinements of a new laryngotracheal prosthesis, the LT Mold™, and the results from a prospectively collected database of 65 patients.

## Materials and methods

### LT-Mold™

**Fig. 1:**
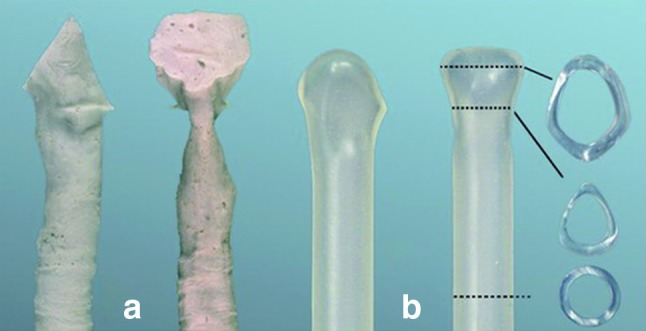
LT-Mold™. **a** Molds of cadaver larynges: the interarytenoid distance is narrow, reflecting the paramedian cadaveric position of the vocal cords. **b** Increased interarytenoid distance of the LT-Mold to restore the triangular shape of the glottis and maintain a large interarytenoid space during the healing process, especially in case of posterior glottic stenosis or cicatricial fusion of the vocal cords

This laryngotracheal prosthesis has been designed for the temporary stenting of the airway after the surgical treatment of a cicatricial stenosis of the larynx. It is made of silicone at a strength of 50 Shores-A. It is soft and thus avoids pressure necrosis at the medial aspect of the arytenoids. Its design was created after molding cadaver larynges and increasing the interarytenoid distance to obtain the intralaryngeal contours of a fully abducted larynx (Fig. 1). This property is essential when treating an SGS combined with a PGS. Since the initial report of a pilot study published in the Laryngoscope in 2003 [14] and our experience in using the LT-Mold™ in 30 pediatric cases published in the International Journal of Pediatric Otorhinolaryngology [15], the LT-Mold has been improved. To avoid possible granulation tissue formation at its distal extremity, a dedicated silicone cap has been manufactured for each size of the prosthesis. The latter exists in 10 different sizes, from 6 to 15 mm in outer diameter and in four different lengths per size. The LT-Mold can be used in pediatric and adult populations and it can be inserted in the airway during open surgery (intraoperative use), or after the endoscopic laser resection of a laryngotracheal stenosis (endoscopic use).

#### Intraoperative use

**Fig. 2:**
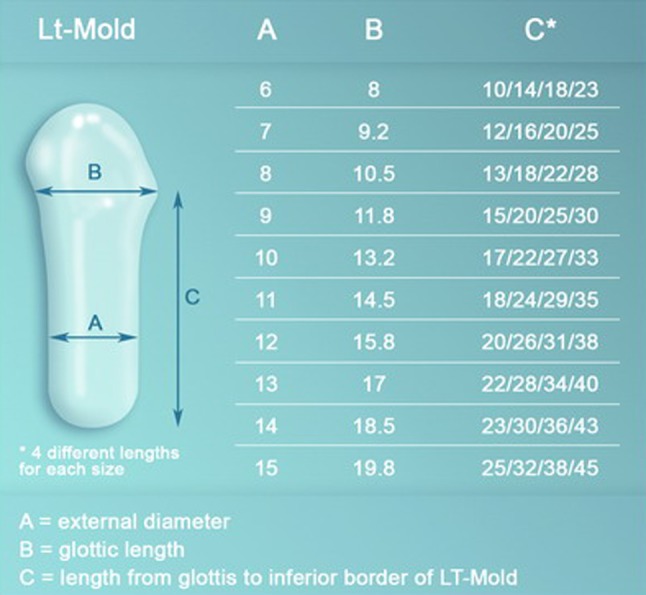
LT-Mold™. Per size, the prosthesis exists in four different lengths to accommodate different positions of the tracheostomy site

During an LTR or a PCTR, the exact distance from the anterior commissure of the larynx to the upper margin of the tracheostoma is measured. With its integrated silicone cap, there is no need for cutting the prosthesis at the appropriate length to secure the cap with glue or stitches during the surgery. Instead, selecting an LT-Mold of appropriate length and diameter is now straightforward with the newly designed prosthesis (Fig. 2). The LT-Mold should be securely fixed to the larynx by placing two non-resorbable (usually 3/0 Prolene) sutures, one through the lateral wall of the trachea at the reinforced cap level of the prosthesis and one in the supraglottis through the ventricular bands. One additional stitch can be precisely placed at the anterior laryngeal commissure with a 5/0 resorbable Vicryl suture to initially secure the prosthesis at the right place.

**Fig. 3:**
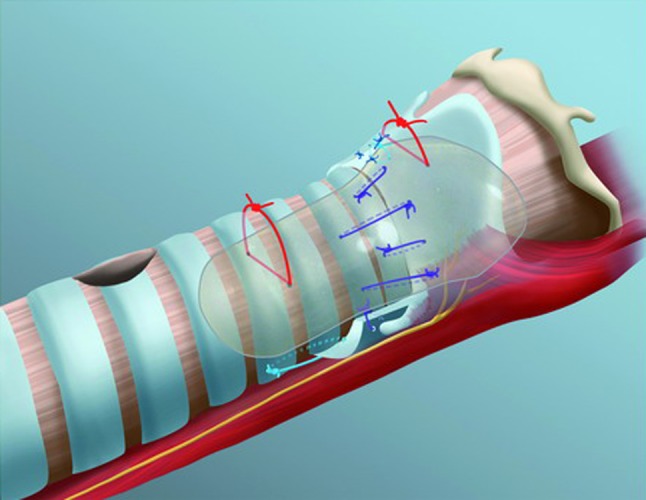
Proper fixation of the LT-Mold™ after extended PCTR. Two 3.0 Prolene transverse stitches (*red*) are placed to fix the prosthesis at the tracheal and supraglottic levels. A 5.0 resorbable Vicryl stitch (in* turquoise*) is additionally placed at the anterior commissure of the larynx to position the prosthesis exactly at the glottis level

**Fig. 4:**
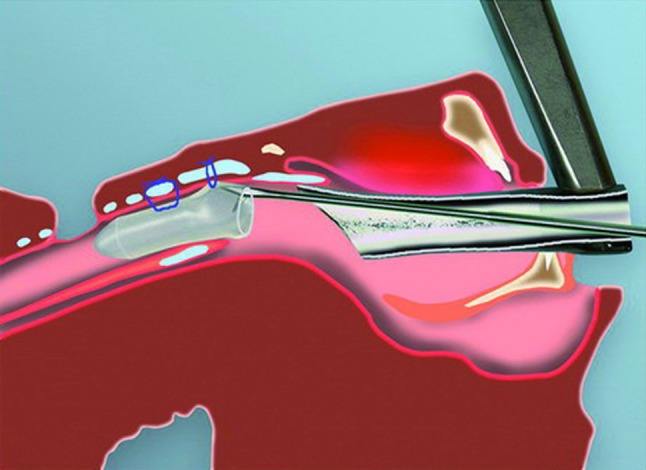
Endoscopic removal of the LT-Mold™. The larynx is exposed with a Lindholm laryngoscope, and the head of the prosthesis is uncapped with curved microscissors. The Prolene fixation threads are cut inside the prosthesis which is removed with an alligator forceps. The Prolene threads are pulled out with a cup forceps

The latter stitch is extremely important, as it will fix the prosthesis exactly at the right level to restore a sharp anterior commissure of the glottis (Fig. 3). During the healing phase, conventional use of a cannula is possible without restriction, a significant advantage in children with small airways. Removal of the prosthesis is performed in suspension micro-laryngoscopy after the appropriate period of stenting has elapsed. With microscissors, the proximal head of the prosthesis is uncapped, and the non-resorbable sutures are cut inside the prosthesis (Fig. 4). Then the prosthesis is gently removed and the non-resorbable sutures are grasped and pulled out with a biopsy forceps. Despite the placement of the knots outside the larynx and trachea during open surgery, it is possible to pull the sutures through into the lumen of the airway in over 90 % of the cases. When this fails, the intraluminal portions of the thread are cut flush with the mucosa and the rest of the non-resorbable threads is left in place.

#### Endoscopic use

**Fig. 5:**
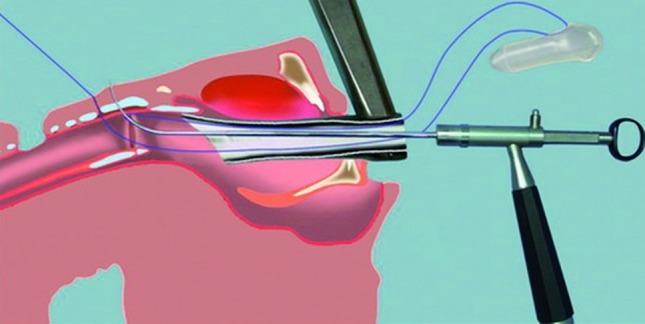
Endoscopic placement of the LT-Mold™ prosthesis in suspension microlaryngoscopy. The 3.0 Prolene stitch is placed first through the prosthesis and then fixed endoscopically with the Lichtenberger needle carrier as described in the text

The LT-Mold can also be used for stenting a glotto-subglottic stenosis that has been resected and/or dilated by endoscopic means. The only prerequisite is the presence of a tracheostoma. In suspension micro-laryngoscopy, the appropriate size prosthesis is inserted through the glottis. The tracheostomy cannula is temporarily removed, and the prosthesis is pushed caudally until its anterior commissure exactly matches that of the larynx. The exact distance from the glottis to the upper margin of the tracheostoma is measured. The appropriate LT-Mold (in length and diameter) is chosen. Endoscopic fixation is done as follows: a stitch using 3.0 Prolene suture material, 70 cm in length, is passed just below the level of the anterior commissure of the prosthesis and retrieved 1 cm caudally. Next, a Lichtenberger needle carrier is used to fix the prosthesis to the larynx in suspended micro-laryngoscopy as shown in Fig. 5. The anterior commissure of the larynx is always easily identified. The distal inside-out stitch is placed first and its insertion site is determined by measuring exactly 1 cm from the anterior commissure of the larynx. This is easily achieved by locating the distance on the shaft of the Lichtenberger needle-carrier at the upper edge of the laryngoscope. The second inside-out stitch is placed just below the anterior commissure of the larynx. Both needle and thread are pulled through the skin and tied over a small dressing placed on the anterior neck. For stenting that will be in place for several weeks, it is preferable to make a small, 1-cm-long horizontal skin incision midway between the points of exit of the threads. With a skin hook, it is easy to recapture the threads under the skin and to tie the knots in the subcutaneous plane. The external fixation is thus buried under the skin, which is then closed in one layer with 5.0 PDS sutures. A final endoscopic check is used to verify the correct position of the prosthesis. The tracheostomy cannula can be introduced and changed without problems when necessary. Removal of the prosthesis after the appropriate time of stenting is performed as described in the section on intraoperative use.

### Patients

After obtaining the approval from our institutional review board, the LT-Mold has been used in 65 patients (36 men and 29 women) with a mean age of 10 years (range 2.5 months to 66 years). There were 53 (81 %) infants and children and 12 (19 %) adult patients. Among pediatric cases, 20 (38 %) were younger than 3 years of age and 39 (36 %) were younger than 6 years of age at the time of surgery. The indications for stenting are shown in the Table 1.

**Table 1:** Indications for stenting with the LT-Mold in 65 cases

Indications for stenting	Nb
Primary surgery	36 (55 %)
Post-inbutation stenosis	21
Glotto-subglottic	14
Posterior glottis stenosis	3
Subglottic stenosis	2
Transglottis stenosis	2
Congenital stenosis	6
Cohen’s Grade IV webbing	4
Elliptical cricoid	2
Other	9
Caustic supraglottic stenosis	3
Intractable aspiration	3
Acute laryngeal trauma	1
Stenotic sequelae of LED	1
Stenotic squelae of RRP	1
Salvage surgery for complex recurrent LTS	29 (45 %)
Failed LTRs	12
Failed PCTRs	4
Inappropriate laser treatment	7
Other endoscopic treatments	6

In 29 (45 %) of the 65 patients, the indication for stenting was a complex recurrent LTS after failed previous surgeries (12 LTRs, 4 PCTRs, 7 inappropriate laser treatments and 6 other endoscopic treatments for 3 laryngeal cleft repairs, 1 persistent tracheoesophageal fistula, 1 caustic injury and 1 papillomatosis, respectively).

Primary surgical interventions with the LT-Mold were performed in 36 (55 %) patients. Thirty-two (89 %) of them suffered from LTS and four patients had no stenosis. The indication for using the LT-Mold in non-stenotic larynges was to protect the lower airway from chronic intractable aspiration of neurological causes in three patients and reconstruction of acute laryngeal trauma in one patient. Other indications included cicatricial supraglottic stenosis induced by caustic ingestion in three cases, transglottic stenoses resulting from laryngeal trauma in two cases and sequelae of lupus erythematosus and papillomatosis in one case, respectively. Severe sequelae of prolonged intubation were found in 20 (63 %) of the 32 patients who sustained severe stenosis (3 isolated PGSs, 2 isolated SGSs, 14 glotto-subglottic stenoses and 1 transglottic stenosis, respectively). Finally six congenital laryngeal stenoses were treated primarily (2 isolated SGSs with elliptical cricoid and 4 glotto-subglottic stenoses, of which 3 were associated with a 90 % webbing of the vocal cords).

According to the modified Myer-Cotton classification [16], there were 4 Grade II (one IIc, three IId); 35 Grade III (one IIIa, one IIIb, twenty-five IIIc, eight IIId); 22 Grade IV (twelve IVc, ten IVd); three patients without stenosis treated for broncho-aspiration and one patient with acute laryngeal trauma. In 55 cases, the LT-Mold was inserted into the larynx during open surgery (28 LTRs, 23 extended PCTRs, 2 PCTRs and 2 surgeries for acute laryngeal trauma and caustic injury, respectively). In 10 cases, the LT-Mold was placed endoscopically after laser reopening of the supraglottis and/or glottis: in 2 cases of pharyngolaryngeal stenosis resulting from caustic injuries, in 4 cases of isolated PGS and in 3 cases of chronic broncho-aspiration, respectively.

The sizes of the LT-Molds ranged from 6 to 14 mm (median 8 mm) in outer diameter, while the lengths ranged for 10 to 36 mm (median 20 mm). Sizes 8 in diameter and 20 in length were by far the most frequently used, in 20 and 17 cases each, respectively.

The duration of stenting was decided upon evaluation of the complexity and steadiness of the airway reconstruction. Its mean value was 3 months (range 1–12 months).

## Results

**Fig. 6:**
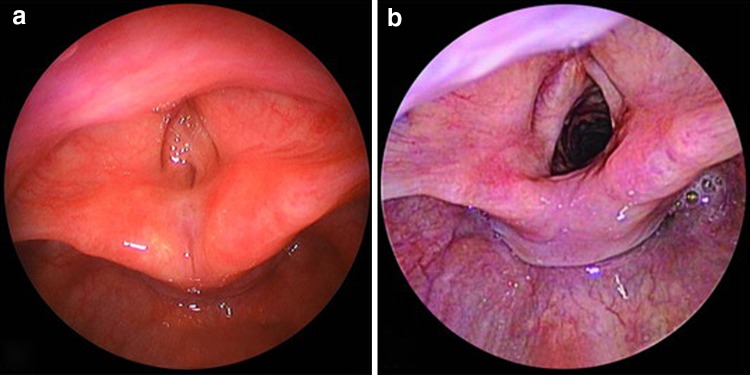
Transglottic Grade IV laryngotracheal stenosis. **a** Total obstruction of the airway at the glottic level, with a supraglottic band of scar tissue tethering the ventricular bands together. The stenosis extended caudally to the inferior edge of the cricoid cartilage. **b** Postoperative view at 3 months after extended-PCTR and stent removal: patent airway with triangular shape of the airway at the glottic level. A stent such as the LT-Mold is necessary to restore a sharp anterior commissure of the larynx after extended-PCTR or LTR for synechia or webbing of the vocal cords

In all 65 patients, the stent has been extremely well tolerated. There has been no erosion or significant granulation tissue formation in the supraglottis and glottis, especially at the medial aspect of the arytenoids. In the 44 cases where a Grade IV or Grade III glotto-subglottic stenosis with vocal cord involvement needed to be addressed, near-normal anterior commissure of the larynx was restored (Fig. 6). In the subglottis, some granulation tissue over cartilage grafts was seen in five patients where the prosthesis was removed somewhat prematurely. In 16 patients, granulation tissue was clearly induced by the lower sharp end of the prosthesis (similar to that of the Montgomery T-tube) at the distal end of the prosthesis. This observation led us to design a round-shaped cap to the distal end of the prosthesis. Since then, we have seen only minor granulation tissue formation at the upper pole of the tracheostoma in the 23 cases that were treated accordingly. However, in three cases we have to report loss of the distal cap of the LT Mold, twice in the supra-tracheostomal region and once in the distal airway. The latter event required bronchoscopic removal of the foreign body. These complications occurred after 4 months of stenting in all three cases. In one of them, the LT-Mold had been inserted endoscopically for intractable broncho-aspiration. These adverse events promoted investigation of the primer and the silicone glue, as well as the potential influence of contamination of the prosthesis with saliva. To avoid further such complications, an integrated cap was glued with a long acting silicone glue to prevent any potential loss of the cap even after long-term stenting.

Fifty-four (83 %) of the 65 patients are presently decannulated with a mean follow-up of 23 months (range 1 month to 10 years) after tracheostoma closure. In one patient suffering from severe broncho-aspiration, the LT-Mold prosthesis is still in place. Three patients died from unrelated complications within the context of severe congenital anomalies. One patient sustained a complete restenosis after loss of the LT-Mold and difficult compliance of the parents who denied further reinsertion of the prosthesis. Another patient could not be decannulated because of mental disability and swallowing problem despite a patent airway. Finally, five patients are awaiting decannulation.

None of the patients required reintervention. The current status of the reconstructed airways is 61 (94 %) of the 65 patients with patent airways (Fig. 6). The subglottic lumen was gauged at 90–100 % patency in all 4 Grade II and 35 Grade III LTS and also in the four patients without cicatricial stenosis (3 chronic broncho-aspirations and one acute laryngeal trauma). Slight recurrence to less than Grade I stenosis was seen in 4 (18 %) of the 22 Grade IV LTS. However, all of these cases were belonging to the group of the most challenging airway problems resulting from failed previous surgeries and severe pharyngo-laryngeal sequelae from caustic injuries.

## Discussion

There is a general consensus on the use of stents in LTR for benign stenoses. They should be avoided unless they are absolutely necessary. This precept is related to the potential risks of complications from indwelling stents. If the prosthesis does not precisely conform to the inner laryngeal contours, if its consistency is too hard or if its extremities have sharp edges, then it can cause mucosal ulceration, granulation tissue formation and restenosis.

We agree with Zalzal [17, 18] that long-term (more than 2 months) stenting is indicated in the following situations: cartilage grafting for absence of rigidity, severely altered anatomy at the glottis level, distorted larynx resulting from previously failed LTR, poor stability of cartilage grafts and posterior cricoid split without grafting. Unfortunately, all currently available stents do not meet the basic requirements for safe use without potential damage to the reconstructed airway. The Aboulker stent is a cigar-shaped prosthesis primarily designed to splint the airway after LTR in children. In the late 1960s, Aboulker [9] reported decannulation in three out of five children after airway reconstruction. Since the 1970s, Grahne [19] and then Cotton [20] and Crysdale [21] also used this stent for stabilizing LTRs in children and reported some good results. Subsequently, other surgeons have used this prosthesis for stenting airway reconstructions [4, 17, 22]. Although the highly polished Teflon of the Aboulker stent is well tolerated by tissues, this prosthesis is too hard and does not conform to the complex inner contours of the larynx. In 1992, Zalzal [18, 23] reviewed all complications linked with the use of the Aboulker stent. He noted granulation tissue formation occurring at the inferior or superior end of the stent, pressure necrosis at the base of the epiglottis and on the medial aspect of the arytenoids, infection, stent migration and broken stents [23, 24]. Furthermore, this cigar-shaped stent cannot restore a sharp anterior commissure of the larynx in case of cicatricial fusion of the vocal cords. This has a negative impact on the quality of the voice.

The Montgomery T-tube is soft and pliable, which allows easy insertion through the tracheostoma [25]. It is well tolerated by the underlying mucosa, but both of its extremities have sharp cut edges that promote granulation tissue formation at the stent-mucosal interface. This occurs preferentially in the conic-shaped subglottis if the upper end of the stent is placed below the vocal cords. It is thus recommended that the upper end of the stent be placed slightly higher than the level of the false vocal cords. Nonetheless, this position can still produce granulation tissue on the laryngeal aspect of the epiglottis or the ventricular bands. To protect the airway from aspiration, the upper extremity of the stent must be closed by a suture or by a plug of silicone glue. Although the Montgomery T-tube can be effective for stenting purely tracheal stenoses, it is far from being ideal for stenting airway reconstructions of the glottis and subglottis [26]. Furthermore, similar to the Aboulker stent, its round-shaped configuration cannot restore a sharp anterior commissure of the glottis. In children, concern exists regarding the safety of the stent when sizes <8 mm in outer diameter must be used. The prosthesis can become plugged with dried secretions. This potentially lethal complication requires prompt removal of the T-tube [27, 28]. Reported complications have included the removal of the T-tube by the child, forward migration resulting in tube expulsion and formation of granulation tissue and plugging [29].

To overcome the risk of clogging of the Montgomery T-tube in children, Healy designed a dedicated pediatric T-tube with a 70° connecting angle that allows the introduction of a flexible inner cannula. Although this pediatric T-tube permits quick removal of plugged secretions in the inner cannula, the latter further diminishes the size of the airway in an already small (<7 mm) T-tube. This pediatric counterpart of adult tracheal stents also shares all of the aforementioned drawbacks of the Montgomery T-tube used in older children and adults.

Currently, only two stents have been designed to preserve or restore endoluminal anatomy of the larynx, but both fail to achieve this goal. The Montgomery LT-stent is made of plain silicone. It is quite hard and was designed after obtaining molds of cadaver larynges, but unfortunately its posterior interarytenoid distance remains that of a cadaveric larynx with the vocal cords in the paramedian position. Thus, it is not appropriate for stenting airway reconstructions for SGS combined with PGS. Furthermore, it only exists in two different sizes, which is largely insufficient to address the whole spectrum of laryngotracheal stenoses in adults and children. Currently, this stent is seldomly used in airway reconstructions [30]. The Eliachar LT-stent is a hollow stent made of soft silicone and is thus less traumatic to the underlying mucosa of the larynx. Initially, it was designed for the management of chronic aspiration [13]. Although its conformity to the inner laryngeal contours is superior to that of all other stents, its shape is not triangular at the level of the glottis. In providing internal support to laryngeal airway reconstructions, it neither restores a large interarytenoid distance nor a sharp anterior commissure of the glottis. Finally, it cannot be used in infants and children, and its fixation system with a silicone strap through the tracheostoma can induce severe granulation tissue formation at the tracheostoma site.

In 1992, Zalzal [17] reviewed all features deemed to be necessary in an ideal stent. He identified five major characteristics: (a) availability in many sizes and shapes to fit reconstructed areas; (b) placement that avoids any risk of obstruction of the respiratory passages; (c) absence of foreign-body reaction, pressure necrosis or discomfort; (d) adequate voice production and easy food intake without aspiration; (e) easy examination and removal. Based upon these requirements, all currently available stents leave much to be desired.

The experience that we have gathered with the LT Mold in 65 patients treated for complex LTS indicates that this new prosthesis is close to meeting all the abovementioned requirements except for voice production. Given that most of these difficult cases result from several previously failed surgeries and often present to the surgeon with aphonia, a further delay of several months until successful decannulation and voice production are restored is quite acceptable.

Because the prosthesis is soft, smooth and pliable, it can easily be inserted into the airway either endoscopically or during open surgery. The key issue is the avoidance of pressure necrosis and granulation tissue formation in the supraglottis, glottis, subglottis and trachea at the tracheostoma site. The design of a silicone integrated cap has helped solve this problem. In all cases treated accordingly, neither granulation tissue formation at both round-shaped extremities of the prosthesis nor suprastomal collapse has been observed. This advantage permits longer term (>6 months) stenting without any risk of further damaging the reconstructed airway. As mentioned by Zalzal [17], long-term stenting increases the risk of granulation tissue formation and potential subsequent restenosis. To avoid extrusion of the prosthesis, which occurred in 14 (21 %) of the 65 cases in this series, tight fixation with non-resorbable (3.0 Prolene) sutures is mandatory. During open surgery, it is recommended that the prosthesis be fixed at the anterior commissure of the larynx with a resorbable transverse stitch and at the suprastomal level with a non-resorbable 3/0 Prolene suture passed through the stent-cap junction which is slightly thicker than the rest of the prosthesis. When fixed by endoscopic means, only one cranio-caudal stitch can be placed with the Lichtenberger needle-carrier, which increases the risk of prosthesis dislodgement. Finally, the LT-Mold can be utilized event in infants, since it does not compromise the conventional use of a tracheotomy cannula.

## Conclusion

The silicone LT-Mold is extremely well tolerated and helps solve the most difficult cases of glotto-subglottic stenoses that require long-term stenting in addition to surgery. When used with its distal integrated cap, the LT-Mold can be left in place for several months without any risk of losing its distal cap. Granulation tissue formation in the supraglottic and glottic area or at the upper margin of the tracheostoma remains minimal, when the prosthesis is correctly placed inside the larynx.

## References

[1] Jaquet Y, Lang F, Pilloud R, Savary M, Monnier P (2005). Partial cricotracheal resection for pediatric subglottic stenosis: long-term outcome in 57 patients. J thorac cardiovasc surg.

[2] White DR, Cotton RT, Bean JA, Rutter MJ (2005) Pediatric cricotracheal resection: surgical outcomes and risk factor analysis. Arch otolaryngol Head Neck surg 131(10):896–899. doi:10.1001/archotol.131.10.896 10.1001/archotol.131.10.896 16230593

[3] Cotton RT, Gray SD, Miller RP (1989). Update of the Cincinnati experience in pediatric laryngotracheal reconstruction. Laryngoscope.

[4] Ndiaye I, van den Abbeele T, Francois M, Viala P, Tanon-Anoh MJ, Narcy P (1999) Surgical management of laryngeal stenosis in children. Annales d’oto-laryngologie et de chirurgie cervico faciale : bulletin de la Societe d’oto-laryngologie des hopitaux de Paris 116(3):143–148 10399529

[5] Ochi JW, Evans JN, Bailey CM (1992). Pediatric airway reconstruction at Great Ormond Street: a ten-year review. I. Laryngotracheoplasty and laryngotracheal reconstruction. Ann Otol Rhinol Laryngol.

[6] Goode RL, Shinn JB (1977). Long-term stenting in the treatment of subglottic stenosis. Ann Otol Rhinol Laryngol.

[7] Evans JN (1977). Laryngotracheoplasty. Otolaryngol Clin North Am.

[8] Aboulker P (1962) Problèmes actuels d’oto-rhino-laryngologies. In: Chirurgie de la trachée. Librairie Maloine, Paris France, p 273

[9] Aboulker P (1968) Treatment of tracheal stenosis. Problemes actuels d’oto-rhino-laryngologie, pp 275–295 5758454

[10] Aboulker P, Sterkers JM, Demaldent JE (1966). Modifications apportées à l’intervention de Rethi, intérêts dans les sténoses laryngo-trachéales et trachéales. Ann Otol Rhinol Laryngol.

[11] Montgomery WW (1965). T-tube tracheal stent. Arch Otolaryngol.

[12] Montgomery WW, Montgomery SK (1990). Manual for use of Montgomery laryngeal, tracheal, and esophageal prostheses: update 1990. Ann otol rhinol laryngol Suppl.

[13] Eliachar I, Nguyen D (1990) Laryngotracheal stent for internal support and control of aspiration without loss of phonation. Otolaryngol Head Neck Surg Official J Am Acad of Otolaryngol Head Neck Surg 103 (5 (Pt 1)):837–840 10.1177/019459989010300531 2126112

[14] Monnier P (2003). A new stent for the management of adult and pediatric laryngotracheal stenosis. Laryngoscope.

[15] Monnier P (2007). Airway stenting with the LT-Mold: experience in 30 pediatric cases. Int J Pediatr Otorhinolaryngol.

[16] Monnier P, Ikonomidis C, Jaquet Y, George M (2009). Proposal of a new classification for optimising outcome assessment following partial cricotracheal resections in severe pediatric subglottic stenosis. Int J Pediatr Otorhinolaryngol.

[17] Zalzal GH (1988). Use of stents in laryngotracheal reconstruction in children: indications, technical considerations, and complications. Laryngoscope.

[18] Zalzal GH (1992). Stenting for pediatric laryngotracheal stenosis. Ann Otol Rhinol Laryngol.

[19] Grahne B (1971). Operative treatment of severe chronic traumatic laryngeal stenosis in infants up to three years old. Acta Otolaryngol.

[20] Cotton RT, Evans JN (1981) Laryngotracheal reconstruction in children. Five-year follow-up. The Annals of otology, rhinology, and laryngology 90 (5 Pt 1):516–520 10.1177/000348948109000522 7305211

[21] Crysdale WS (1983). Subglottic stenosis in children. A management protocol plus surgical experience in 13 cases. Int J Pediatr Otorhinolaryngol.

[22] April MM, Marsh BR (1993). Laryngotracheal reconstruction for subglottic stenosis. Ann Otol Rhinol Laryngol.

[23] Zalzal GH, Grundfast KM (1988). Broken Aboulker stents in the tracheal lumen. Int J Pediatr Otorhinolaryngol.

[24] Mohr RM (1992). A modification of the Aboulker stent for reduction of granulation tissue that allows tracheotomy changes. Laryngoscope.

[25] Montgomery WW (1974). Silicone tracheal T-tube. Ann Otol Rhinol Laryngol.

[26] Cooper JD, Todd TR, Ilves R, Pearson FG (1981). Use of the silicone tracheal T-tube for the management of complex tracheal injuries. J Thorac Cardiovasc Surg.

[27] Stern Y, Willging JP, Cotton RT (1998). Use of Montgomery T-tube in laryngotracheal reconstruction in children: is it safe?. Ann Otol Rhinol Laryngol.

[28] Calhoun KH, Deskin RW, Bailey BJ (1988). Near-fatal complication of tracheal T-tube use. Ann Otol Rhinol Laryngol.

[29] Froehlich P, Truy E, Stamm D, Floret D, Morgon A (1993). Role of long-term stenting in treatment of pediatric subglottic stenosis. Int J Pediatr Otorhinolaryngol.

[30] Montgomery WW (1968). The surgical management of supraglottic and subglottic stenosis. Ann Otol Rhinol Laryngol.

